# Urotropine in Biliary and Pancreatic Inflammations

**Published:** 1908-05-23

**Authors:** 


### UROTROPINE IN BILIARY AND PANCREATIC INFLAMMATIONS.

It is well enough known that hexa-methylene-tetramine (urotropine) is a valuable antipyuric remedy, its beneficial action in cases of cystitis and pyelitis depending apparently upon the excretion of formalin in the urine. It is less generally known that the products of the drug appear not only in the urine, but also in the secretions of other glands besides the kidney, and notably in the bile and in the pancreatic juice. Those who have been familiar with these facts have used the drug in the treatment of various inflammatory affections of the bile and pancreatic ducts, with highly satisfactory results in a great many cases.

Just as in the urine, so in the bile and pancreatic juice, the amount of formalin present when urotropin is administered by the mouth must necessarily be very small, and the fluids cannot be compared to any surgical lotion as regards active bactericidal properties. It is apt to be forgotten, however, that a dilution of antiseptic which is a hundredfold and more too weak to destroy the life of micro-organisms may yet succeed in inhibiting their rate of multiplication. If the check to multiplication can only make the number of new germs produced in a given time less than the number washed out from the biliary or pancreatic passages by the flow of the secretions in the same time, cure results.

The excretion of hexamethylenamin in the bile and pancreatic juice of dogs was determined by Mr. Crowe in the following manner:—After exposure of the duodenum an opening was made a little over an inch below the pylorus. This incision exposed the orifices of the pancreatic and common bile ducts, which in dogs open separately. The bile and pancreatic juice were collected by means of small catheters inserted into the ducts.

The experiments on dogs all showed that hexamethylenamin is excreted in the bile and pancreatic juice. In one case it was found to be present in the bile contained in the gall-bladder twenty-four hours after giving 15 grains of the drug by the mouth, and it could also be demonstrated in the pancreatic juice obtained under the action of secretin. The strength of the formalin was estimated as equivalent to 1 in 10,000. Further experiments showed that the drug is secreted not only by the hepatic cells, but also by those in the walls of the gall-bladder itself.

The following experiment showed that urotropine is rapidly absorbed, and remains in the circulating blood for some hours. A rabbit was given 7 grains by the mouth, and a quarter of an hour later 1 c.c. of blood removed from an ear-vein gave a decisive test. A faint trace was still present in the blood removed twenty-four hours after giving the drug. Apparently the maximum concentration in the blood is reached in 5 to 8 hours after the drug is given.

The following is one of several illustrative cases given by Mr. Crowe:—The patient was a married woman, aged 45, under the care of Dr. Kelly in Johns Hopkins Hospital, admitted for some pelvic trouble. During the routine exploration at the time of the operation the gall-bladder was discovered to be distended with gall-stones. A second incision was made, and forty-five small calculi, together with much sandy mucoid material, were removed and a biliary fistula made. Ten days later the patient received her first dose of hexamethylenamin. Within the next twenty-hours she had five doses of 15 grains each.

Some of the material obtained from the sinus was tested, and showed that either hexamethylenamin or its decomposition product, formaldehyde, was present in the bile in quantity. It was sufficient to inhibit entirely the growth of organisms in the cultures made.

It is clear, therefore, that hexamethylenamin (urotropine) has a wide field of usefulness, not only in urinary diseases, but also in such other affections as:—(1) Acute and sub-acute infections of the gall-bladder; of the bile ducts, large and small; of the pancreatic ducts. (2) Possibly in some cases of mastitis. (3) During convalescence from typhoid fever, where it would act not only as a prophylactic measure against the subsequent formation of gall-stones, but also by sterilising the gall-bladder and thus preventing the patient from becoming a "typhoid carrier." (4) Before gall-bladder operations, as a prophylactic measure.

